# Mediastinal Hibernoma: A Rare Case with Radiologic-Pathologic Correlation

**DOI:** 10.1155/2016/2378143

**Published:** 2016-08-29

**Authors:** Maxine Darke, Anil Dasyam, Matthew Then, Kavita Varma, Amir A. Borhani, Rakesh Varma

**Affiliations:** University of Pittsburgh Medical Center, 200 Lothrop Street, Pittsburgh, PA 15213, USA

## Abstract

Hibernomas, especially located in the mediastinum, are extremely rare benign tumors, which are important to consider in the differential diagnosis of a heterogeneously enhancing mass with areas of fat attenuation on imaging of an often incidentally discovered mass. Other common possibilities in the differential include malignant tumors, such as liposarcoma, hence histopathology is usually required to confirm the diagnosis. Hibernomas often follow the distribution of sites of persistence of brown fat in adults, and intrathoracic locations are unusual. We present a very rare case of a mediastinal hibernoma in a 53-year-old woman. She presented to the emergency department with severe, progressive right neck and shoulder pain with radiation down her arm and was found to have a right apical posterior mediastinal mass on imaging. Initial radiographs of the shoulder showed a soft tissue mass within the apical right hemithorax. Further imaging with CT revealed a well circumscribed, heterogeneously enhancing mass with areas of fat attenuation. Pathology confirmed the diagnosis of mediastinal hibernoma, and the mass was completely excised. Fourteen months after surgery, the patient had a normal chest radiograph, and thirty-two months after surgery, she remains asymptomatic.

## 1. Introduction

Hibernomas are rare, benign tumors composed of brown fat. Hibernomas can occur in multiple sites, including the head, neck, trunk, and extremities. Only a few mediastinal hibernomas have previously been documented in literature. These tumors enhance heterogeneously and often contain macroscopic fat, which can result in misdiagnosis as liposarcoma. While imaging can provide useful clues, the diagnosis needs histopathologic confirmation. We present a very rare case of a mediastinal hibernoma with excellent radiology-pathology correlation.

## 2. Case Presentation

A 53-year-old woman with no significant past medical history presented to the emergency department with a ten-day history of progressive, severe right neck and shoulder/scapular pain that was worse with movement and radiated down her right arm to her fingers. NSAIDs, range of motion exercises, and trigger point injections had provided only minimal relief. She denied recent trauma, had no infectious symptoms, and reported no smoking history.

**Figure 1:**
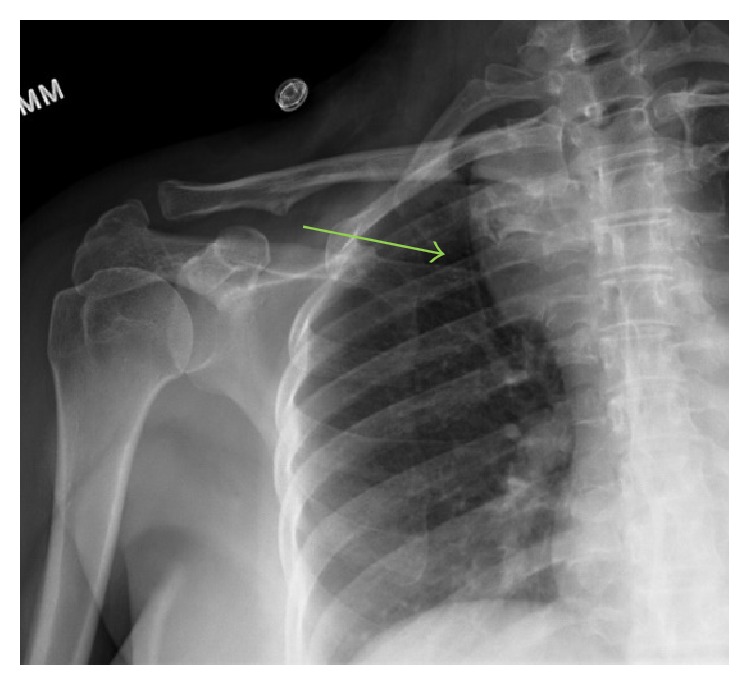
Frontal radiograph of the right shoulder shows a superior mediastinal mass (green arrow) within the right hemithorax.

**Figure 2:**
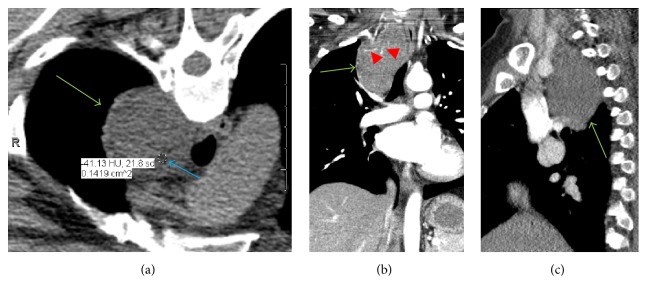
Contrast enhanced axial CT (a) with coronal (b) and sagittal (c) CT reconstructions of the chest demonstrating a well circumscribed, heterogeneously enhancing mass (green arrows) with areas of fat attenuation (blue arrow pointing to ROI in (a)) and a few mildly prominent internal vessels (red arrow heads in (b)) within the superior, posterior right mediastinum.

**Figure 3:**
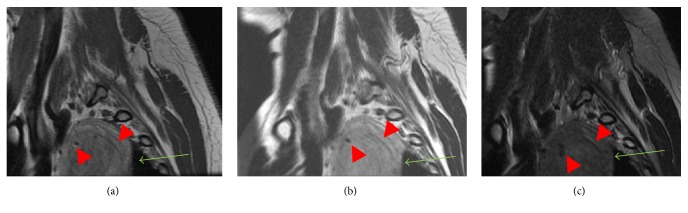
Sagittal T1 (a), T2 (b), and fat saturation T2 (c) weighted images from the cervical spine MRI show a heterogeneous, T1, and T2 hyperintense mass (green arrows in (a) and (b)) with a few internal flow voids (red arrow heads in (a), (b), and (c)) and moderate loss of signal with fat saturation (green arrow in (c)).

Right shoulder radiographs revealed a right superior mediastinal mass (Figure 1). Further imaging with contrast enhanced CT showed a 7.2 cm well demarcated, heterogeneously enhancing mass with areas of fat attenuation and some prominent internal vessels within the right superior, posterior mediastinum (Figure 2). The mass anteriorly abutted the brachiocephalic artery and trachea but did not invade adjacent structures. A cervical spine MRI obtained for cervical radiculopathy symptoms partially included the mass, which was heterogeneous, hyperintense to muscle on both T1 and T2 weighted sequences with a few internal flow voids, and showed moderate loss of signal with fat saturation (Figure 3).

**Figure 4:**
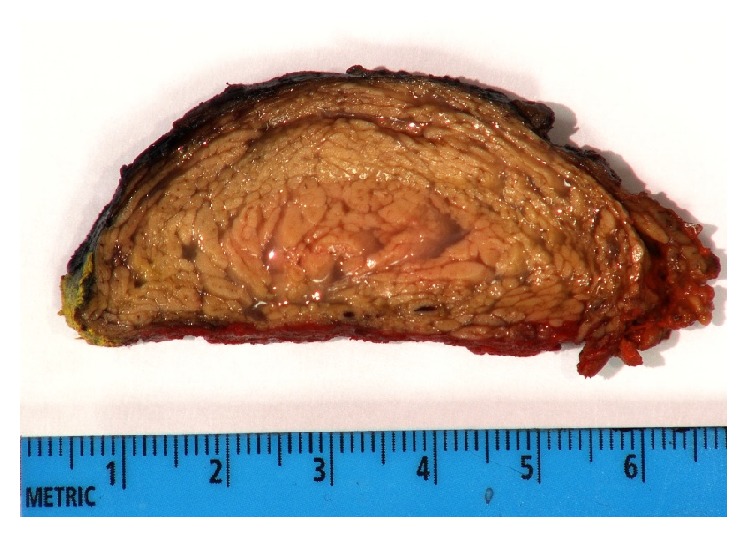
The cut surface of the gross specimen shows a circumscribed, yellow to tan, lobulated mass.

**Figure 5:**
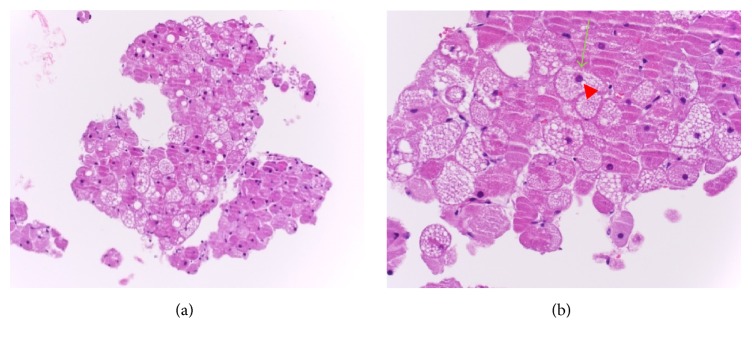
H&E, 40x (a) and 200x (b), respectively, demonstrating sheets of cells resembling brown fat. The higher power examination revealing well demarcated granular, multivacuolated eosinophilic cells (green arrow in (b)) with small centrally placed nuclei (red arrow head in (b)).

Mesenchymal neoplasms, lymphoma, and teratoma were considered in the differential diagnosis based on imaging. Microscopically, the CT-guided biopsy showed multivacuolated and granular eosinophilic cells with small centrally located nuclei consistent with hibernoma. No lipoblasts or cytologic atypia was seen to suggest liposarcoma. Due to the unusual location, large size, and possible mass effect on adjacent structures, total excision was performed by a right video-assisted thoracoscopy. The surgeon described a dense, fatty, lobulated mass located in the posterior mediastinum with extension into the lower neck and without direct invasion of surrounding structures. The resection specimen measured 8 × 5 × 3 cm. Grossly, it was described as a circumscribed, yellow to tan, lobulated mass (Figure 4). Histologic sections demonstrated cytologically bland brown fat cells with abundant granular cytoplasm identical to the preoperative biopsy (Figure 5). Due to their classic histomorphologic features, most hibernomas can readily be diagnosed without the use of ancillary studies. Cytogenetic studies have demonstrated rearrangements of 11q13; however, amplification of MDM2 should prompt consideration of an atypical lipomatous tumor/well differentiated liposarcoma. The patient was discharged on the second postoperative day.

On follow-up, the patient had transient right vocal cord paralysis, likely due to the close proximity of the resected mass to the recurrent laryngeal nerve, with return of full vocal cord mobility about one month after surgery. No CT evidence of recurrence was seen on a chest CT four months after surgery, and a chest radiograph fourteen months after surgery was normal. Thirty-two months after surgery, the patient remains asymptomatic.

## 3. Discussion

Tumors composed of brown fat are called hibernomas, a term that originated due to the similarity to brown fat in hibernating animals [1]. Hibernomas are rare, benign, and slow growing and are often asymptomatic incidental findings on imaging or physical exam [2, 3].

Since these tumors are hypothesized to arise from tiny remnants of fetal brown fat, it is logical that common sites for hibernomas are areas known to contain brown fat in adults [1, 3]. In the largest review of 170 hibernomas, the most common locations were the thigh and the shoulder. Intrathoracic hibernomas, including the mediastinum and pleura, only accounted for 0.065% (11/170) of those cases [4]. In 2004, only the fourth mediastinal hibernoma was reported in the world literature [3]. Therefore, our case of a hibernoma in the posterior mediastinum is extremely rare and demonstrates excellent radiologic-pathologic correlation.

It is important to recognize hibernomas from imaging and pathology as benign tumors, not to be confused with malignant tumors like liposarcomas. On CT, hibernomas are well circumscribed, heterogenous lesions with mixed low attenuation fatty components and enhancing soft tissue components. It is essential to note that hibernomas are hypervascular, a key imaging differentiation from liposarcomas [2, 5]. On MRI, hibernomas are often hyperintense on T1 and T2 weighted images with loss of some signal on fat suppression images; striking contrast enhancement and flow voids on T2 weighted images are other MR features that can be identified [1, 5]. Despite the tumor's hypervascularity, Papathanassiou et al. reported no complications from core needle biopsy in their series [6].

Gross pathology is typically described as a tan colored, well-defined, soft, lobulated mass that can be encapsulated [2]. Histology commonly reveals multivacuolated fat cells that contain small centrally located nuclei; atypia should not be present [4]. The tan color of the mass is due to the combination of hypervascularity and high mitochondria content. The four histologic variants include typical, which is most common, myxoid, lipoma-like, and spindle cell [2]. Positive staining for S-100 is a typical, but nonspecific, feature with 85% (17/20) of cases in one study reported as positive [4]. Pathologic features that are worrisome for liposarcoma include lack of vascularity, cellular atypia, and irregular septa [1].

Although the masses are most commonly asymptomatic, they can grow to large sizes and become symptomatic. Therefore, treatment with complete surgical resection is common. Incomplete excision can lead to local recurrence of the tumor [6]. Overall, prognosis is excellent. There are no known cases of hibernoma metastases or malignant transformation [2, 5].

## References

[1] da Motta A. C., Tunkel D. E., Westra W. H., Yousem D. M. (2006). Imaging findings of a hibernoma of the neck. *American Journal of Neuroradiology*.

[2] Murphey M. D., Carroll J. F., Flemming D. J., Pope T. L., Gannon F. H., Kransdorf M. J. (2004). Benign musculoskeletal lipomatous lesions. *Radiographics*.

[3] Baldi A., Santini M., Mellone P. (2004). Mediastinal hibernoma: a case report. *Journal of Clinical Pathology*.

[4] Furlong M. A., Fanburg-Smith J. C., Miettinen M. (2001). The morphologic spectrum of hibernoma: a clinicopathologic study of 170 cases. *The American Journal of Surgical Pathology*.

[5] Little B. P., Fintelmann F. J., Mino-Kenudson M., Lanuti M., Shepard J.-A. O., Digumarthy S. R. (2011). Intrathoracic hibernoma: a case with multimodality imaging correlation. *Journal of Thoracic Imaging*.

[6] Papathanassiou Z. G., Alberghini M., Taieb S., Errani C., Picci P., Vanel D. (2011). Imaging of hibernomas: a retrospective study on twelve cases. *Clinical Sarcoma Research*.

